# Low Back Pain in Italian Nurses: A Statistical Analysis of Disability and Work Productivity Impairment—An Observational Study

**DOI:** 10.3390/healthcare13091016

**Published:** 2025-04-28

**Authors:** Roberto Lupo, Elsa Vitale, Luana Conte, Andrea Bernetti, Francesco Ciccarese, Marcella Orgiu, Salvatore Latina, Ludovica Panzanaro, Alessia Lezzi, Alessandra Puglia, Giorgio De Nunzio, Donato Cascio, Gianandrea Pasquinelli, Ivan Rubbi

**Affiliations:** 1 “San Giuseppe da Copertino” Hospital, Local Health Authority (ASL) of Lecce, 73100 Lecce, Italy; roberto.lupo@asl.lecce.it; 2 Oncologia Medica per la Patologia Toracica, IRCCS Istituto Tumori “Giovanni Paolo II”, 70124 Bari, Italy; vitaleelsa00@gmail.com; 3 Department of Physics and Chemistry, University of Palermo, 90128 Palermo, Italy; donato.cascio@unipa.it; 4 Laboratory of Interdisciplinary Research Applied to Medicine (DReAM), University of Salento and Local Health Authority (ASL) of Lecce, 73100 Lecce, Italy; giorgio.denunzio@unisalento.it; 5 Department of Experimental Medicine (Di.Me.S), University of Salento, 73100 Lecce, Italy; andrea.bernetti@unisalento.it; 6 Infradepartmental University Program of Physical and Rehabilitation Medicine, “V.Fazzi” Hospital, Local Health Authority (ASL) of Lecce, 73100 Lecce, Italy; 7 “San Giuseppe” RSA Carmiano, 73041 Lecce, Italy; francesco.ciccarese.bn@gmail.com; 8 School of Nursing, Local Health Authority (ASL) Romagna, University of Bologna, 48018 Faenza, Italy; marcella.orgiu@studio.unibo.it (M.O.); ivan.rubbi@auslromagna.it (I.R.); 9 “Umberto I” Hospital, 96014 Siracusa, Italy; latinasalvo@gmail.com; 10 C.R.A.P. Carrubo, Sol Levante Srl, Avetrana, 74020 Taranto, Italy; ludovica.panzanaro@hotmail.it; 11 ANT Italia ONLUS Foundation (National Cancer Association), 73100 Lecce, Italy; 1alessia.lezzi@gmail.com; 12 “A. Perrino” Hospital, Local Health Authority (ASL) of Brindisi, 72100 Brindisi, Italy; alessandra.puglia@live.it; 13 Department of Mathematics and Physics, University of Salento, 73100 Lecce, Italy; 14 Department of Medical and Surgical Sciences, University of Bologna, 40126 Bologna, Italy; gianandr.pasquinelli@unibo.it

**Keywords:** low back pain, nurses, Quebec Back Pain Disability Scale, Oswestry Disability Index, Work Productivity and Activity Impairment Questionnaire

## Abstract

**Background**: Low back pain (LBP) is a common occupational health issue among nurses, significantly affecting quality of life and work productivity. Despite awareness, it remains a major cause of absenteeism and presenteeism, highlighting the need for targeted interventions. This study aimed to assess the prevalence of LBP among Italian nurses and its impact on quality of life and work productivity. **Methods**: A cross-sectional, multicenter observational study was conducted from May to October 2024 using an online questionnaire distributed to members of the Provincial Orders of Nursing Professions across Italy. The questionnaire included sociodemographic variables and three validated instruments: the Quebec Back Pain Disability Scale (QBPDS), the Oswestry Disability Index (ODI), and the Work Productivity and Activity Impairment Questionnaire (WPAI). **Results**: A total of 318 nurses participated, with the majority from Southern Italy (57.1%) and female (74.6%). LBP was reported by 57.5% of respondents. Nurses working in Critical Care and those with 30–40 years of experience had significantly higher QBPDS and ODI scores, indicating moderate disability. Nurses working 12 h shifts and those with job restrictions or medical prescriptions reported significantly higher disability levels (ODI > 29, *p* < 0.001). Nurses on pharmacological therapy reported moderate pain levels, while those engaging in regular physical activity had significantly lower pain symptoms (<20, *p* < 0.001). The WPAI results showed that 67.0% of nurses reported impaired work productivity due to LBP. **Conclusions**: LBP is extremely prevalent among Italian nurses, especially affecting physical well-being and, accordingly, the health care quality provided by them. Factors exacerbating this problem are wrong manual handling of loads, not exercising, poor nutrition, and smoking, as well as wrong posture. Fundamental in order to avoid the occurrence of this problem are preventive programs and ergonomic training.

## 1. Introduction

Low back pain (LBP) is a significant public health issue affecting both the general population and workers, particularly those in the healthcare sector [1]. Estimates indicate that by 2050, at least 843 million people will be affected by LBP. In Europe, LBP is considered one of the leading causes of workplace absenteeism and productivity loss [2,3]. In Italy, the situation is similarly concerning, with a high prevalence among the adult population, significantly impacting the quality of life and overall well-being [2,4]. Several occupational factors play a crucial role in the development of LBP, especially in work environments that require frequent lifting, prolonged postures, and repetitive movements [2,5]. The healthcare sector, in particular, has a high incidence of LBP due to the significant physical and mental stress experienced by healthcare workers [2,6]. These risk factors not only increase the likelihood of developing LBP but also complicate its management, necessitating targeted interventions tailored to the specific workplace context [7,8,9].

Back pain is highly prevalent among nurses, with reports indicating a frequency of nearly 90%. LBP in nurses is mainly associated with lifting heavy loads during patient handling, transfers, and manual mobilization. However, this accounts for only 10% of cumulative lumbar compression, whereas approximately 80% originates from other caregiving activities that lead to poor postures during procedures, prolonged standing, and weight-free ambulation [10]. The scientific literature extensively documents the incidence of LBP in this professional group, emphasizing its negative impact on nurses’ ability to provide adequate patient care and its association with increased absenteeism [2,11,12].

Nurses over the age of forty show a higher prevalence of LBP compared with those under the age of thirty-one. Aging is associated with reduced muscle capacity; stretching, atrophy, and muscle weakening contribute to discomfort as professionals grow older [13,14]. LBP has also been found to be more frequent among female nurses than among their male counterparts. Although the exact cause is unknown, this may be related to anatomical, physiological, and structural differences [15], as well as to non-occupational activities that women are more often engaged in, such as domestic tasks [16]. A correlation between LBP and marital status was also observed. Married nurses experience a higher rate of LBP than single nurses, especially those with large families and multiple children [13]. Regarding professional workload, it was noted that nurses working more than seven hours a day have a higher risk of developing LBP [17]. This appears to be due to repeated exposure to strenuous tasks that often strain the lumbar region. Additionally, nurses frequently have to work extra hours to cover for absent colleagues or due to staff shortages. Psychophysical fatigue, lack of rest due to increased working hours, and stress all seem to significantly contribute to the occurrence of LBP among nursing staff [18,19]. Another key factor in the development of LBP is the manual handling of loads, which is often carried out without proper equipment and with inadequate staff training [13,18,20].

The implications of LBP extend beyond physical discomfort. This condition significantly affects workplace safety, particularly in occupations that involve frequent physical exertion and improper postures. LBP can severely compromise workers’ ability to perform their duties effectively and safely. Other consequences include impaired well-being, reduced job satisfaction, and decreased overall quality of life. Acute episodes of LBP can become chronic and disabling, leading to workforce reduction and burnout [10]. Studies have demonstrated a strong correlation between LBP and the onset of depressive symptoms [21], creating a negative cycle in which physical pain exacerbates psychological distress and vice versa. As chronicity progresses, LBP contributes to disability, restricted movement, poorer healthcare outcomes, and increased absenteeism or presenteeism.

Presenteeism, in which employees continue to work despite being unwell, poses an even greater risk, as it exponentially increases the probability of workplace injury and worsens the worker’s condition, ultimately raising healthcare costs [22]. In response to this issue, European and Italian regulations have developed specific guidelines for LBP prevention [23]. These guidelines promote ergonomic interventions and safe work practices, as highlighted in the review by Wallwork SB et al. [24], to prevent the onset of chronic pain.

A key aspect of LBP management involves identifying and addressing associated risk factors, which include age, sedentary lifestyle, obesity, and genetic predisposition [2,5]. Healthcare institutions can mitigate LBP risk through training in manual handling techniques, providing lifting equipment, assigning specialized teams, and implementing workplace training to improve communication with patients, encouraging their cooperation [10]. Workplace initiatives that encourage employees to report early symptoms have been effective in reducing absenteeism by enabling timely intervention [25]. Despite the growing awareness of LBP among nurses, the Italian literature on this topic remains limited, particularly within the Italian healthcare system.

The aim of the study is to determine the prevalence of low back pain among Italian nurses in relation to sociodemographic factors and to analyze its impact on work productivity.

## 2. Materials and Methods

### 2.1. Study Procedures and Data Collection Tool

A cross-sectional, multicenter observational study was conducted from May to October 2024 through the online dissemination of a questionnaire. The starting time of this study was 15 April 2024. The survey was distributed via a link sent to all the presidents of the Provincial Orders of Nursing Professions nationwide (n = 102). The presidents of the Orders of Nursing Professions across the country were contacted and divided into four geographical areas: Northwest Italy (Liguria, Lombardy, and Piedmont), Northeast Italy (Emilia-Romagna, Friuli-Venezia Giulia, Trentino-Alto Adige, and Veneto), Central Italy (Lazio, Marche, and Tuscany), and Southern Italy (Abruzzo, Basilicata, Calabria, Campania, Molise, Puglia, Sicily, and Sardinia). The Orders of Nursing Professions in the provinces of Lecce, Varese, Ancona, La Spezia, Latina, and Potenza participated in this study. An email was sent to all provincial order presidents introducing the study and formally requesting participation in the survey. After obtaining approval from the presidents, each nurse belonging to the respective order received an email containing a brief introduction to the study and a link to access the online questionnaire. Data collection and analysis methods were designed to ensure participant anonymity. Each nurse was required to complete the questionnaire in full and completely anonymously. The orders participating in the study strongly recommended that each nurse complete the questionnaire only once to avoid selection bias and prevent redundant data collection. The questionnaire was always completed in the same order by all participants, and the response mode required participants to answer all questions.

### 2.2. Description of the Survey Tool

The questionnaire used for data collection was structured into two main sections. The first section collected sociodemographic information, including age, gender, years of experience, work area, and work schedule distribution. These variables were essential for analyzing potential correlations between demographic factors and LBP prevalence. The questions were formulated clearly and concisely to facilitate comprehension and ensure accurate responses.

The second section of the questionnaire comprises three validated instruments in the Italian language. The Quebec Back Pain Disability Scale QBPDS [26] was used to assess how LBP affects participants’ daily activities. Each question was rated on a scale from 0 to 5, generating an overall score that reflects the perceived level of disability. This tool has been widely used in the literature and is considered one of the most reliable for evaluating LBP-related disability. Another instrument used was the Oswestry Disability Index (ODI) 2.1, refs. [26,27], which is designed to assess the degree of disability associated with back pain. The questionnaire includes questions on various aspects, such as personal care, weight lifting, walking ability, and sleep quality. Participants were required to select only one answer per question, contributing to an overall score that measures the impact of LBP on daily life. The ODI is internationally recognized and frequently used in clinical research.

The final tool incorporated was the Work Productivity and Activity Impairment Questionnaire (WPAI), ref. [28], which focuses on the impact of LBP on work productivity and daily activities. The questions assess absenteeism, health-related productivity impairment, and the effect of LBP on daily activities. The responses provided by participants allow for quantification of the level of impairment due to LBP. This tool is particularly useful for understanding the economic impact of the condition, both at an individual level and within healthcare organizations.

All instruments were digitized using the Google Forms platform, ensuring a user-friendly interface that increased the likelihood of participation and minimized the risk of data entry errors. Nurses were able to complete the questionnaire in a secure and private environment, contributing to improved response quality.

### 2.3. Inclusion and Exclusion Criteria

All nurses belonging to the Orders of Nursing Professions who had been granted permission to participate in the study and were included in the mailing list of each provincial order were recruited. Nurses who voluntarily agreed to participate, including nursing coordinators and managers working in both public and private sectors, were included. Nursing professionals awaiting their first employment were excluded. Nurses with back pain with pathologies related to trauma or tumors were excluded.

### 2.4. Data Analysis

The database was created using Microsoft Excel (version 2503), while statistical analyses were conducted using Jamovi 2.3.18. Descriptive statistical analyses (frequencies, percentages, means, medians, and standard deviations) were performed, and significance levels were determined using ANOVAs and *t*-tests. Internal consistency was assessed using Cronbach’s alpha, while sample adequacy was evaluated using the Kaiser–Meyer–Olkin (KMO) test.

### 2.5. Ethical Considerations

Before participating in the study, nurses were provided with clear information regarding the study’s objectives, the nature of their participation, potential risks and benefits, and the handling of personal data. A detailed description of the study was provided to ensure that participants could make an informed decision. Participation was voluntary, and nurses had the right to withdraw from the study at any time without any negative consequences. This voluntary participation policy was essential to ensure that only those who felt comfortable sharing their experiences took part in the research. The study received ethical approval from the Ethics Committee of the University of Bologna (Protocol No. 0102302, 10 April 2024).

## 3. Results

### Sociodemographic Characteristics of the Sample

As described in Table 1, a total of 318 professionals from across the country responded to the questionnaires, with the majority coming from Southern Italy (57.1%, n = 181). Female participants accounted for 74.6% (n = 235), and the age distribution, divided into ten-year intervals from 21 to 60 years, maintained a proportional representation of approximately 20% per age group. The vast majority of respondents (92.1%) were in nursing/midwifery roles and provided direct patient care. Regarding educational qualifications, 79.4% (n = 250) held only the basic professional qualification. In terms of work areas, 22.5% (n = 71) worked in Critical Care, 20.6% (n = 65) in Surgical Units, 19.4% (n = 61) in Multi-Specialty Medical Areas, 14.3% (n = 45) in Geriatric-Rehabilitation, 13.3% (n = 42) in Community-Based Nursing, 6.7% (n = 21) in Organization and Training, and 3.2% (n = 10) in Maternal-Childcare Units.

Regarding work experience, 24.1% (n = 76) of respondents had between 1 and 5 years of experience, followed by 18.7% (n = 59) in the 30–40-year category, 16.2% (n = 51) with 6 to 10 years of experience, with the remaining categories each representing less than 15%. The vast majority (96.8%, n = 305) worked full-time, while 61.3% (n = 193) worked in 24 h shift rotations. Regular shift schedules were reported by 52.7% (n = 166).

Regarding clinical characteristics, the sample had an average height of 1.67 ± 10.3 cm and a mean weight of 70.9 ± 15.8 kg. A total of 64.8% (n = 204) were smokers, 60.3% (n = 190) were on pharmacological therapy, and 56.2% (n = 177) engaged in physical activity. Among respondents, 81.0% (n = 255) reported work-related limitations, 67.0% (n = 211) were at risk for manual material handling (MMH) at work, 68.9% (n = 217) had attended a course on proper MMH techniques, and 57.5% (n = 181) reported experiencing LBP.

Table 2 shows very good internal consistency and an adequate sample size for the QBPDS and ODI. The instruments produced average scores corresponding to mild disability in both assessments.

Table 3 highlights statistically significant differences in disability scores between participants with and without LBP. Professionals who reported experiencing LBP had an average QBPDS score of 19.01 ± 14.00 and an ODI score of 19.84 ± 14.17 compared with scores below 5 in those without LBP (*p* < 0.001). These differences were further confirmed by the WPAI which indicated that, although limited, work productivity was compromised in individuals with LBP (*p* < 0.001). A similar trend was observed in responses regarding the extent to which health problems affected daily activities.

Nurses affected by LBP reported significantly higher QBPDS and ODI scores among female participants compared with males, as shown in Table 4. Notably, ODI scores in female participants indicated a moderate level of disability, with a mean exceeding 20. In terms of age, LBP severity was moderate in both instruments, with higher scores observed in the 51–60 and >60-year age groups. Years of work experience also had a significant impact, with 30–40 years of experience associated with moderate disability scores > 20.

Among work areas, nurses in organizational, managerial, and training roles reported significantly higher LBP scores, exceeding 30 in both QBPDS and ODI. This outcome is likely the result of previous career transitions from other areas where professionals had experienced incompatibility between their health conditions and the physical demands of their roles.

Twelve-hour shifts were associated with significantly higher disability scores than twenty-four-hour shifts, possibly due to medical recommendations regarding shift assignments. Regular shifts also had significantly higher scores (>20, *p* < 0.05). Nurses with work restrictions or medical prescriptions reported significantly higher pain scores (>29).

Regarding clinical characteristics and lifestyle habits, participants on pharmacological therapy reported moderate pain levels, while regular physical activity was associated with significantly lower pain symptoms (<20).

## 4. Discussion

The present study examined the prevalence of LBP among Italian nurses, revealing concerning results that are consistent with international research. The prevalence rates in our sample, exceeding 50%, align with previous studies. For instance, Brusini et al. [5] reported annual incidence rates ranging from 13.7% to 20% and prevalence rates between 17% and 63.7%. They identified night shifts, insufficient training, frequent patient handling, lack of equipment, work units, obesity, age, work-related stress, and physical inactivity as major risk factors for LBP among Italian nurses.

In our sample, lumbar pain appears to be consistent with the WHO 2023 report. Pain symptoms are particularly prevalent among middle-aged nurses (aged 51–60) and older nurses, with a higher prevalence in females, as indicated by an average ODI score above twenty. These findings suggest a correlation between LBP and disability, with significant differences between affected and unaffected healthcare workers.

Among the study participants, several nurses reported mild disability, a condition that could expose professionals to work limitations and, consequently, decreased productivity. However, our data did not reveal significant absenteeism due to pain, a finding also confirmed in the literature [29]. The continued presence of nurses at work despite painful symptoms may not only compromise their overall well-being but also lead to possible disability and chronicity of the condition [5,10]. Even though absenteeism was not documented in our sample, LBP and its related adverse effects could increase the risk of absenteeism to the point of leading nurses to leave the profession altogether [10]. The lack of recorded absenteeism may be explained by the presence, among participants with LBP, of attitudes documented in literature as “introverted”, “exploitable”, and “subassertive”, which push nurses to show up for shifts despite health issues [30], thus increasing the likelihood of injury or health deterioration [31].

Our study highlights an increased prevalence of LBP among nurses with 16 to 20 years of work experience. It is particularly observed in those working in organizational, managerial, or educational areas, as well as among nurses working 12 h shifts compared to those working 24 h shifts, despite the literature identifying night shifts as a risk factor for LBP [5,32]. These data may reflect an incompatibility between health status and the physical demands of certain roles. Nurses with disabilities may be reassigned or request assignment to lower-stress areas, where occupational control is achieved through technical, organizational, and individual interventions [33].

Excluding these situations, among nurses in our sample suffering from LBP, the most affected work areas are Critical Care, Multidisciplinary Medical, Surgical, and Community Health Services—settings characterized by more frequent manual load handling. These findings are consistent with the existing literature [21].

The data also suggest that medications are moderately effective in pain control, but physical activity appears to be a stronger protective factor against LBP, with significant reductions in painful symptoms [34]. Among nurses with LBP, those who reported engaging in physical activity had QBPDS and ODI scores below 20.

Physical exercise helps strengthen muscles and improve physical performance and posture, which is crucial during nursing care activities. Poor posture, prolonged standing, unbalanced walking, and heavy lifting have all been identified as contributing factors [13,33,34]. Over 55% of participants reported engaging in physical activity, including 100 out of 181 participants affected by LBP. However, since we did not quantify or classify the type of physical activity, this constitutes a study limitation.

Notably, despite 68.9% of participants having attended a course on Manual Load Handling (MLH), a substantial portion of nurses still report low back pain as they continue to work in high-risk environments for load handling. This suggests that, despite training, other factors may influence LBP onset. Inadequate practical application of training, improper posture, prolonged standing, and other caregiving activities that do not involve lifting may still lead to lumbar strain [13,18,20].

A strength of our study is the use of validated assessment tools, including the QBPDS, the ODI, and the modified WPAI. These instruments proved useful in evaluating the impact of LBP on nurses’ daily lives and work. Although our findings mostly concern nurses with mild to moderate functional and occupational disabilities, they highlight the need for targeted interventions. One such intervention is the Back School program, which has shown to be effective in managing chronic non-disabling LBP. It includes exercises, multidisciplinary therapeutic programs, behavioral therapy, and NSAID use. This comprehensive approach aims to educate individuals on how to maintain correct posture and proper movement across all aspects of life, including the workplace. It serves as both a preventive and rehabilitative strategy to reduce pain through progressively more complex and targeted training. In general, maintaining physical activity—such as walking, moving, and avoiding static positions—is essential. Ergonomic modifications at home and in the workplace are also critical [35]. For nurses, this includes proper seating, ergonomic stations for computer use, adjustable beds to avoid straining during procedures, appropriate footwear for prolonged standing, and availability of lifting equipment [10,36].

Manual load-handling training is also a valuable strategy for reducing and preventing LBP among nurses. However, such knowledge is often not well retained or applied. Therefore, it would be appropriate to implement more frequent and periodic training sessions to safeguard workers’ health [37].

## 5. Study Limitations

Despite its significant findings, our study has some limitations. First, the sample size may not fully represent the entire Italian nursing population. Additionally, the sample consisted primarily of relatively young nurses with less than ten years of experience, limiting the generalizability of the findings to older or more experienced nurses.

Other limitations include reliance on self-reported data, which may introduce subjective bias, the absence of longitudinal follow-up, which would allow for an analysis of LBP progression over time, and the lack of investigation into whether or not the back pain among the sample analyzed was related to any sort of spine pathology. Future research should aim to include larger and more diverse samples and further explore gender dynamics and the impact of socioeconomic variables on LBP among nurses.

## 6. Conclusions

This study demonstrates a clear correlation between the nursing profession and low back pain (LBP) among Italian nurses, with a prevalence exceeding 50% among participants. Moderate disability was especially observed among nurses aged 51–60 and those over 60 years old. LBP is more common among female nurses and appears to be highly disabling, particularly on a physical level, as it limits daily activities and, most importantly, work performance. As a result, physical limitations at work prevent nurses from fully performing their professional duties. Consequently, many nurses are forced to request transfers or are reassigned to lower-demand areas such as administrative, managerial, or educational roles.

An alternative strategy appears to be the reduction of working hours, as full-time work with 24 h rotating shifts is also identified as a contributing factor to the onset of LBP. This study acknowledges that the number of years of service has a significant impact on LBP risk, with a higher prevalence among those working in high-demand clinical areas such as critical care, multidisciplinary medical units, surgical departments, and community health services. These settings clearly require considerable physical effort.

Physical activity is confirmed as a protective factor, significantly reducing pain, with ODI scores falling below 20. Regular exercise, a healthy lifestyle, abstaining from smoking, and balanced nutrition may all contribute to lowering the risk of LBP. Furthermore, although 68.9% of participants reported attending a course on manual load handling (MLH), many were still affected by LBP. This highlights how several nursing work environments remain at risk for MLH-related injuries and how the application of acquired knowledge in practice may be inadequate or insufficiently retained.

For this reason, a potential solution could be to increase the frequency and regularity of MLH training sessions, along with broader educational efforts on this topic, in order to protect healthcare workers’ health—and, by extension, that of patients, who are the ultimate recipients of quality care provided by professionals.

## Figures and Tables

**Table 1:** Participant characteristics.

	n	%
Sociodemographics		
Geographical Area		
North	101	32.1
Center	33	10.5
South—Islands	181	57.5
Gender		
Female	235	74.6
Male	80	25.4
Age Group		
21–30	76	24.1
31–40	80	25.4
41–50	60	19.0
51–60	87	27.6
>60	12	3.8
Job Role		
Nursing/Midwife	290	92.1
Nursing Coordinator/Manager	25	7.9
Education Level		
Bachelor’s degree or equivalent	250	79.4
Postgraduate training	65	20.6
Work Area		
Critical Care and Emergency	71	22.5
Geriatric-Rehabilitation	45	14.3
Multi-specialty medical	61	19.4
Surgical	65	20.6
Maternal-Child	10	3.2
Community-based	42	13.3
Organizational/Training	21	6.7
Years of Experience		
1–5	76	24.1
6–10	51	16.2
11–15	39	12.4
16–20	27	8.6
21–25	21	6.7
26–30	42	13.3
30–40	59	18.7
Work Schedule		
Full-time	305	96.8
Part-time	10	3.2
Shift Type		
Day shift (12 h)	122	38.7
24 h shift	193	61.3
Regular Shift		
No	149	47.3
Yes	166	52.7
Weekly Work Hours		
<12–24	19	6.0
25–32	46	14.6
>32	250	79.4
CLINICAL DATA		
	M ± SD	
Weight (kg)	70.9 ± 15.8	
Height (cm)	1.67 ± 10.3	
	n	%
Smoking Status		
No	204	64.8
Yes	86	27.3
Ex	25	7.9
Under medication		
No	190	60.3
Yes	125	39.7
Engages in physical activity		
No	138	43.8
Yes	177	56.2
Work limitations		
No	255	81.0
Yes	60	19.0
Exposure to MMC risk in the workplace		
No	104	33.0
Yes	211	67.0
Attended a training course on MMC		
No	98	31.1
Yes	217	68.9
Suffers from low back pain		
No	134	42.5
Yes	181	57.5

M ± SD = Mean ± Standard Deviation; MMC = Manual movement of loads.

**Table 2:** Overall results, internal consistency, and sample size of the instruments.

	n. Item	Range	M ± SD	Median	α	KMO
Quebec Back Pain Disability Scale (QBPDS)	20	0–100	12.8 ± 14.9	8.00	0.964	0.952
Oswestry Disability Index (ODI)	10	0–50	13.2 ± 13.8	10	0.917	0.942

α = Cronbach’s alpha; M ± SD = Mean ± Standard Deviation; KMO = Kaiser–Meyer–Olkin.

**Table 3:** Results of disability assessment tools in the sample with and without low back pain.

	No Low Back Pain		Yes, Low Back Pain			
	n = 134		n = 181			
	M ± SD	Median	M ± SD	Median	t	*p*
QBPDS	4.64 ± 6.81	3.00	19.01 ± 14.00	14.00	−9.580	<0.001 **
ODI	4.62 ± 6.86	2.00	19.84 ± 14.17	18.00	−11.470	<0.001 **
WPAI						
Hours of work absence in the last 7 days	0.56 ± 3.42	0.00	0.82 ± 3.57	0.00	−0.670	0.503
Hours worked in the last 7 days	33.35 ± 11.30	36.00	32.68 ± 10.00	36.00	0.550	0.582
To what extent have health problems affected productivity in the last 7 days?	1.34 ± 2.00	0.00	3.06 ± 2.62	3.00	−6.340	<0.001 **
To what extent have health problems affected normal daily activities (excluding work) in the last 7 days?	1.31 ± 2.02	0.00	3.56 ± 2.55	4.00	−8.444	<0.001 **

M ± SD = Mean ± Standard Deviation; ** *p* ≤ 0.01.

**Table 4:** Comparison of disability assessment tools among individuals with low back pain.

		QBPDS		ODI	
	n	M ± DS	*p*	M ± DS	*p*
Gender					
Male	39	13.2 ± 12.8	0.012 *	14.0 ± 11.2	0.003 **
Female	142	20.5 ± 16.8		21.4 ± 14.5	
Age Group					
21–30	24	12.9 ± 11.1	<0.001 **	14.4 ± 9.47	<0.001 **
31–40	35	10.2 ± 6.98		12.7 ± 11.3	
41–50	41	19.0 ± 15.4		19.6 ± 12.1	
51–60	71	24.5 ± 18.8		24.6 ± 14.4	
>60	10	25.5 ± 17.7		24.8 ± 22.9	
Years of Work Experience					
1–5	28	12.9 ± 10.6	<0.001 **	14.3 ± 9.56	<0.001 **
6–10	23	11.3 ± 7.65		14.3 ± 11.8	
11–15	16	11.1 ± 8.62		11.5 ± 9.65	
16–20	18	16.7 ± 16.7		15.1 ± 10.3	
21–25	16	22.1 ± 16.3		24.4 ± 12.4	
26–30	31	18.5 ± 13.7		21.6 ± 11.0	
30–40	49	28.9 ± 20.2		27.5 ± 17.6	
Work Area					
Critical Care Area	36	13.8 ± 12.1	0.008 **	17.7 ± 11.5	0.010 *
Geriatric-Rehabilitation Medical Area	22	21.0 ± 14.6		21.9 ± 12.1	
Multi-specialty Medical Area	34	20.8 ± 16.3		19.7 ± 15.2	
Surgical Area	35	15.1 ± 15.5		14.8 ± 11.6	
Maternal-Child Area	6	11.7 ± 9.65		16.7 ± 9.18	
Community Healthcare Area	32	21.4 ± 15.9		21.6 ± 13.7	
Organizational, Managerial, and Training Area	16	30.9 ± 23.8		31.0 ± 20.8	
Work Shifts					
12 h shift	84	23.8 ± 17.8	<0.001 **	24.4 ± 16.0	< 0.001 **
24 h shift	97	14.9 ± 13.8		15.9 ± 11.0	
Shift Regularity					
Irregular	71	15.6 ± 13.1	0.026 *	16.5 ± 10.1	0.009 **
Regular	110	21.2 ± 17.8		22.0 ± 15.9	
Hours Worked per Week					
<12–24	10	23.9 ± 11.4	0.327	22.6 ± 8.80	0.796
25–32	29	21.9 ± 19.9		20.3 ± 13.9	
>32	142	18.1 ± 15.7		19.6 ± 14.5	
Takes Medication					
No	86	14.7 ± 12.2	<0.001 **	15.1 ± 9.57	<0.001 **
Yes	95	22.9 ± 18.5		24.2 ± 16.2	
Physical Activity					
No	81	22.0 ± 17.4	0.025 *	23.1 ± 14.8	0.005 **
Yes	100	16.6 ± 15.1		17.2 ± 13.1	
Work Limitations					
No	126	14.5 ± 12.5	<0.001 **	15.5 ± 10.2	<0.001 **
Yes	55	29.4 ± 19.2		29.7 ± 16.8	
Exposure to MMC Risks					
No	50	22.0 ± 16.5	0.125	21.2 ± 15.3	0.442
Yes	131	17.9 ± 16.2		19.3 ± 13.7	
Training on MMC					
No	60	16.8 ± 16.2	0.198	17.5 ± 11.8	0.123
Yes	121	20.1 ± 16.4		21.0 ± 15.1	

M ± SD = Mean ± Standard Deviation; MMC = Manual movement of loads; * *p* ≤ 0.05; ** *p* ≤ 0.01.

## Data Availability

Anonymized data that support the findings of this study are available from the corresponding author upon reasonable request.

## References

[1] Vlaeyen J.W.S., Maher C.G., Wiech K., Van Zundert J., Meloto C.B., Diatchenko L., Battié M.C., Goossens M., Koes B., Linton S.J. (2018). Low Back Pain. Nat. Rev. Dis. Prim..

[2] Russo F., Di Tecco C., Fontana L., Adamo G., Papale A., Denaro V., Iavicoli S. (2020). Prevalence of Work Related Musculoskeletal Disorders in Italian Workers: Is There an Underestimation of the Related Occupational Risk Factors?. BMC Musculoskelet. Disord..

[3] Ferreira M.L., de Luca K., Haile L.M., Steinmetz J.D., Culbreth G.T., Cross M., Kopec J.A., Ferreira P.H., Blyth F.M., Buchbinder R. (2023). Global, Regional, and National Burden of Low Back Pain, 1990–2020, Its Attributable Risk Factors, and Projections to 2050: A Systematic Analysis of the Global Burden of Disease Study 2021. Lancet Rheumatol..

[4] Caldo D., Bologna S., Conte L., Amin M.S., Anselma L., Basile V., Hossain M.M., Mazzei A., Heritier P., Ferracini R. (2023). Machine Learning Algorithms Distinguish Discrete Digital Emotional Fingerprints for Web Pages Related to Back Pain. Sci. Rep..

[5] Brusini A. (2021). Low Back Pain among Nurses in Italy: A Review. G. Ital. Med. Lav. Ergon..

[6] Choobineh A., Museloo B.K., Ghaem H., Daneshmandi H. (2021). Investigating Association between Job Stress Dimensions and Prevalence of Low Back Pain among Hospital Nurses. Work.

[7] Vitale E., Conte L., Dell’Aglio A., Calabrò A., Ilari F., Bardone L., Benedetto A., Caldararo C., Zacchino S., Lezzi A. (2021). Healthcare Workers Perceptions in the Difficult Moment of the End of Life and Coping Strategies Adopted during the COVID-19 Pandemic: An Italian Pilot Study. Acta Biomed..

[8] Lupo R., Zaminga M., Carriero M.C., Santoro P., Artioli G., Calabrò A., Ilari F., Benedetto A., Caslini M., Clerici M. (2020). Eating Disorders and Related Stigma: Analysis among a Population of Italian Nursing Students. Acta Biomed..

[9] Conte L., Lupo R., Lezzi A., Paolo V., Rubbi I., Rizzo E., Carvello M., Calabrò A., Botti S., De Matteis E. (2024). A Nationwide Cross-Sectional Study Investigating Adherence to the Mediterranean Diet, Smoking, Alcohol and Work Habits, Hormonal Dynamics between Breast Cancer Cases and Healthy Subjects. Clin. Nutr. Open Sci..

[10] Tariq R.A., George J.S., Ampat G., Toney-Butler T.J. (2025). Back Safety.

[11] Yokota J., Fukutani N., Nin K., Yamanaka H., Yasuda M., Tashiro Y., Matsushita T., Suzuki Y., Yokota I., Teramukai S. (2019). Association of Low Back Pain with Presenteeism in Hospital Nursing Staff. J. Occup. Health.

[12] Schmidt D.C., Rambo T.d.R., Silveira E.C., Wagner L.E., Renner J.D.P., Paiva D.N. (2024). Association between Low Back Pain Perception and Occupational in Hospital Nursing Professionals. Rev. Bras. Med. Trab..

[13] Ayane D., Takele A., Feleke Z., Mesfin T., Mohammed S., Dido A. (2023). Low Back Pain and Its Risk Factors Among Nurses Working in East Bale, Bale, and West Arsi Zone Government Hospitals, Oromia Region, South East Ethiopia, 2021 –Multicenter Cross-Sectional Study. J. Pain Res..

[14] Novielli P., Romano D., Magarelli M., Diacono D., Monaco A., Amoroso N., Vacca M., De Angelis M., Bellotti R., Tangaro S. (2024). Personalized Identification of Autism-Related Bacteria in the Gut Microbiome Using Explainable Artificial Intelligence. iScience.

[15] Balling H., Holzapfel B.M., Böcker W., Simon D., Reidler P., Arnholdt J. (2024). Musculoskeletal Dimension and Brightness Reference Values in Lumbar Magnetic Resonance Imaging—A Radio-Anatomic Investigation in 80 Healthy Adult Individuals. J. Clin. Med..

[16] Hoy D., Bain C., Williams G., March L., Brooks P., Blyth F., Woolf A., Vos T., Buchbinder R. (2012). A Systematic Review of the Global Prevalence of Low Back Pain. Arthritis Rheum..

[17] Carriero M.C., Conte L., Calignano M., Lupo R., Calabrò A., Santoro P., Artioli G., Caldararo C., Ercolani M., Carvello M. (2021). The Psychological Impact of the Coronavirus Emergency on Physicians and Nurses: An Italian Observational Study. Acta Biomed..

[18] Ibrahim M.I., Zubair I.U., Yaacob N.M., Ahmad M.I., Shafei M.N. (2019). Low Back Pain and Its Associated Factors among Nurses in Public Hospitals of Penang, Malaysia. Int. J. Environ. Res. Public Health.

[19] Hämmig O. (2020). Work- and Stress-Related Musculoskeletal and Sleep Disorders among Health Professionals: A Cross-Sectional Study in a Hospital Setting in Switzerland. BMC Musculoskelet. Disord..

[20] Aydın Sayılan A. (2021). Low Back Pain and Methods of Coping with Low Back Pain in Nurses. Ağrı-J. Turk. Soc. Algol..

[21] Nassar N., Assaf N., Farrag D., Ibrahim D., Al-Sheekh A. (2019). Depression in Patients with Chronic Low Back Pain. Egypt. Rheumatol. Rehabil..

[22] Latina R., Petruzzo A., Vignally P., Cattaruzza M.S., Vetri Buratti C., Mitello L., Giannarelli D., D’Angelo D. (2020). The Prevalence of Musculoskeletal Disorders and Low Back Pain among Italian Nurses: An Observational Study. Acta Biomed..

[23] European and Italian regulations Guidelines for Low Back Pain Prevention. https://www.normattiva.it/atto/caricaDettaglioAtto?atto.dataPubblicazioneGazzetta=2008-04-30&atto.codiceRedazionale=008G0104.

[24] Wallwork S.B., Braithwaite F.A., O’Keeffe M., Travers M.J., Summers S.J., Lange B., Hince D.A., Costa L.O.P., Menezes Costa L.d.C., Chiera B. (2024). The Clinical Course of Acute, Subacute and Persistent Low Back Pain: A Systematic Review and Meta-Analysis. Can. Med. Assoc. J..

[25] Bellosta-López P., Blasco-Abadía J., Belsué Pastora J., Hoegh M.S., Palsson T.S., Christensen S.W.M.P., Berjano P.L., Langella F., Vanni D., de Brito Silva P. (2022). Linee Guida Di Buona Pratica per La Gestione Del Dolore e i Disturbi Muscolo-Scheletrici Nei Lavoratori e Nelle Aziende.

[26] Monticone M., Baiardi P., Ferrari S., Foti C., Mugnai R., Pillastrini P., Vanti C., Zanoli G. (2009). Development of the Italian Version of the Oswestry Disability Index (ODI-I). Spine.

[27] van Hooff M.L., Spruit M., Fairbank J.C.T., van Limbeek J., Jacobs W.C.H. (2015). The Oswestry Disability Index (Version 2.1a). Spine.

[28] Lambert J., Hansen B.B., Arnould B., Grataloup G., Guillemin I., Højbjerre L., Strandberg-Larsen M., Reilly M.C. (2014). Linguistic Validation into 20 Languages and Content Validity of the Rheumatoid Arthritis-Specific Work Productivity and Activity Impairment Questionnaire. Patient-Patient-Centered Outcomes Res..

[29] Bozic A., Gajdobranski D., Brestovacki-Svitlica B., Medic-Pericevic S., Mikov M., Vasovic V., Mikov I. (2022). The Prevalence of Low Back Pain among Nurses in Serbia. Work.

[30] Borys C., Nodop S., Anders C., Tutzschke R., Scholle H.C., Thomas A., Altmann U., Strauss B. (2018). Interpersonal Problem Behavior and Low Back Pain. PLoS ONE.

[31] Kwon S., Lee S.J. (2023). Underreporting of Work-related Low Back Pain among Registered Nurses: A Mixed Method Study. Am. J. Ind. Med..

[32] June K.J., Cho S. (2011). Low Back Pain and Work-related Factors among Nurses in Intensive Care Units. J. Clin. Nurs..

[33] Cargnin Z.A., Schneider D.G., Vargas M.A.d.O., Machado R.R. (2019). Dor Lombar Inespecífica e Sua Relação Com o Processo de Trabalho de Enfermagem. Rev. Lat. Am. Enferm..

[34] Indrayani N.L.D., Kao C.-Y., Suyasa I.G.P.D., Padmalatha K.M.S., Chang J.-H., Wang C.-J. (2024). Effectiveness of Exercise Programs to Reduce Low Back Pain among Nurses and Nursing Assistants: A Systematic Review and Meta-Analysis. J. Safety Res..

[35] Negrini S., Romano M., Bardoscia Q. (2005). Lombalgia e Lavoro: Il Contributo Della Riabilitazione. Stato Dell’arte. G. Ital. Med. Lav. Ergon..

[36] Lupo R., Zacchino S., Caldararo C., Calabrò A., Carriero M.C., Santoro P., Carvello M., Conte L. (2020). The Use of Electronical Devices and Relative Levels of Nomophobia within a Group of Italian Nurses: An Observational Study. Epidemiol. Biostat. Public Health.

[37] Berardinelli D. Lombalgia, Programma Formativo per Ridurne La Comparsa. https://www.nurse24.it/dossier/dolore/lombalgia-infermieri-formazione-prevenzione.html.

